# Efficacy of Monotherapy Letrozole Versus Methotrexate for the Management of Ectopic Pregnancy: A Systematic Review and Meta-Analysis of Comparative Studies

**DOI:** 10.3390/jcm14186523

**Published:** 2025-09-17

**Authors:** Ahmed Abu-Zaid, Mohannad Alsabban, Ahmed Nazer, Safa Alabdrabalamir, Mohammed Ziad Jamjoom, Saad M. S. Alqarni, Hedaya Albelwi, Saeed Baradwan, Seham Mabrouk Ebeid, Mohammed Abuzaid, Afnan Baradwan, Osama Alomar

**Affiliations:** 1 Department of Biochemistry and Molecular Medicine, College of Medicine, Alfaisal University, Riyadh 11533, Saudi Arabia; 2 Department of Obstetrics and Gynecology, King Faisal Specialist Hospital and Research Center, Riyadh 11211, Saudi Arabia; 3 Department of Obstetrics and Gynecology, King Fahad Armed Forces Hospital, Jeddah 23311, Saudi Arabia; 4 Department of Obstetrics and Gynecology, King Faisal Armed Forces Hospital, Khamis Mushait 62413, Saudi Arabia; 5 Department of Obstetrics and Gynecology, College of Medicine, Tabuk University, Tabuk 47512, Saudi Arabia; 6 Department of Obstetrics and Gynecology, King Faisal Specialist Hospital and Research Center, Jeddah 23433, Saudi Arabia; 7 Department of Obstetrics and Gynecology, Security Forces Hospitals Program, General Directorate of Medical Services, Ministry of Interior, Riyadh 11134, Saudi Arabia; 8 Department of Obstetrics and Gynecology, Al Birk General Hospital, Al Birk 63525, Saudi Arabia; 9 Department of Obstetrics and Gynecology, Al Salama Hospital, Jeddah 22233, Saudi Arabia

**Keywords:** letrozole, methotrexate, ectopic pregnancy, beta-human chorionic gonadtropin, anti-Mullerian hormone, meta-analysis

## Abstract

**Background**: Ectopic pregnancy (EP) is a serious condition often treated with methotrexate. Letrozole, a safer aromatase inhibitor, may offer an effective alternative. This study presents a meta-analysis comparing the efficacy and safety of single-agent letrozole versus methotrexate for EP management. **Methods**: A systematic review and meta-analysis were conducted following PRISMA guidelines. Six sources of information underwent screening until 12 June 2025. Risk of bias and evidence certainty of evidence were assessed. Primary outcome was treatment success rate. Results were presented as mean difference (MD) or risk ratio (RR) along with a 95% confidence interval (CI) using a random-effects model. **Results**: Six studies (three randomized controlled trials and three nonrandomized prospective cohort studies) comprising seven arms and 260 patients (letrozole = 130, methotrexate = 130) were included. Almost all studies (n = 5) had overall moderate or high risk. Treatment success rates were comparable between groups (n = 7 arms; RR = 1.05; 95% CI: [0.94, 1.17]; *p* = 0.40). Letrozole was associated with significantly lower β-hCG levels on day 4 (n = 5 arms; MD = −95 mIU/mL; 95% CI: [−189.7, −0.91]; *p* = 0.048), day 7 (n = 5 arms; MD = −86.24 mIU/mL; 95% CI: [−143.1, −29.36]; *p* < 0.001), and day 14 (n = 3 arms; MD = −9.15 mIU/mL; 95% CI: [−17.24, −1.06]; *p* = 0.03); however, the differences were not clinically meaningful. Letrozole showed a better safety profile with higher platelet counts and lower liver enzymes. AMH levels were similar between groups. Most analyses were consistent, though secondary outcomes were less stable. Overall evidence certainty was rated ‘very low’ due to seriousness of risk of bias and imprecision. **Conclusions**: While letrozole shows comparable efficacy to methotrexate and a potentially better safety profile in the management of EP, the certainty of evidence is ‘very low’ due to risk of bias and imprecision. Therefore, these findings should be interpreted with caution, and further high-quality studies are urgently needed to confirm the results.

## 1. Introduction

Ectopic pregnancy (EP) occurs when the embryo implants and develops outside the uterine cavity [1]. The fallopian tube is the most common site for extrauterine implantation (95%), although other locations (5%) such as interstitium, ovaries, cervix, abdomen, and within caesarean section scars have also been reported [2]. EP is a lethal condition, particularly if undiagnosed, as it can rupture, leading to severe hemoperitoneum and resulting in maternal mortality in up to 10% of cases [3].

The management of EP depends on the patient’s clinical condition, the size and location of the ectopic pregnancy, and whether the patient is stable. Treatment options include surgical and medical interventions. Surgical interventions such as laparoscopy or laparotomy are indicated for more severe or advanced cases. On the other hand, medical management is effective for early, uncomplicated cases [2,3,4].

Among medical therapies, methotrexate, a chemotherapeutic agent, is the most commonly used treatment for early, unruptured ectopic pregnancies [5]. It works by inhibiting the formation of tetrahydrofolate, which disrupts DNA synthesis and leads to the death of rapidly proliferating trophoblastic cells [6]. Methotrexate has a success rate of up to 90% [5], but it may cause serious side effects that can negatively affect the quality of life and future ovarian reserve [7].

Recent, though limited, studies have suggested that letrozole may offer a role in the medical management of EP [8]. It works by inhibiting aromatase enzyme, leading to reduction in placental estrogen, progesterone, and vascular endothelial growth factor (VEGF) levels, all of which are crucial to the maintenance of the proliferating trophoblastic cells [9,10,11,12].

Several studies have compared the single-agent efficacy of letrozole versus methotrexate for the management of EP [13,14,15,16,17,18]. However, the findings have been limited by a small number of studies, varying study designs, and conflicting conclusions. In 2024, Tarafdari et al. [19] and Laganà et al. [20] conducted two independent meta-analyses based on only two studies. Both studies were constrained by the small number of studies included, as well as the absence of additional analyses such as sensitivity analysis and evaluation of evidence certainty. Moreover, some studies were disregarded while newer ones were published, emphasizing the necessity for a holistic synthesis of all available evidence (now totaling six studies with seven interventional arms) to provide robust, up-to-date recommendations.

The aim of this research is to perform a contemporary and thorough meta-analysis, of all studies comparing single-agent letrozole to methotrexate for the medical management of EP.

## 2. Methods

### 2.1. Study Protocol

This unregistered study in the International Prospective Register of Systematic Reviews (PROSPERO) was conducted in accordance with the steps provided in the Cochrane Handbook for Systematic Reviews of Interventions [21], the Meta-analysis Of Observational Studies in Epidemiology (MOOSE) [22], and the Preferred Reporting Items for Systematic Reviews and Meta-Analyses (PRISMA) statement [23].

### 2.2. Eligibility Criteria

We considered studies that aligned with the following conditions: (i) patients were diagnosed with EP irrespective of the site, (ii) the experimental group received letrozole, (iii) the control group received methotrexate, (iv) the primary endpoint of success rate of EP treatment was reported, and (v) the study design was original comparative studies, inclusive of both retrospective and prospective ones. We excluded studies that did not meet these criteria, such as non-original studies, those using non-pharmacological agents or combination therapies (e.g., letrozole plus methotrexate), and studies comparing medical therapy with surgical interventions (e.g., salpingectomy).

### 2.3. Literature Search

We performed searches in five data sources: Web of Science, PubMed, Scopus, Cochrane Central Register of Controlled Trials, and Google Scholar. The specific search strategy used includes the following query: (letrozole OR femara) AND (methotrexate OR amethopterin) AND (ectopic OR extrauterine OR tubal) AND (pregnancy). The exact search strategy used in all databases is depicted in Supplementary Table S1. No filters were applied in the search, including restrictions based on language, publication date, or location. The search covered records from the inception of each database up to 12 June 2025. After removing duplicates, we screened abstracts and titles to exclude irrelevant citations. We then conducted a full-text review to determine the final studies for inclusion in our analysis. To minimize the chance of missing relevant studies, we also examined the reference lists of included RCTs and recent reviews. The database search was performed independently by two coauthors, with any disagreements resolved through agreement.

### 2.4. Data Collection and Outcomes

We collected data on author names, publication year, country, study design, EP size and site, sample size, mean participant age, mean body mass index (BMI), and details of treatment interventions. The primary endpoint was the success rate of medical treatment for ectopic pregnancy, defined by stable clinical signs, no increase in adnexal mass or hemoperitoneum on ultrasound, a beta-human chorionic gonadotropin (β-hCG) decline of 15% or more or becoming undetectable by day 7, and no need for surgical intervention. The secondary endpoints included the mean β-hCG levels at various time points after medical treatment (days 4, 7, and 14), anti-Müllerian hormone (AMH) levels, and specific side effects indicated by changes in blood parameters such as platelet count and liver enzyme levels. Two teams, each consisting of two coauthors, independently gathered the data and resolved discrepancies through internal consensus.

### 2.5. Assessment of Study Quality and Certainty of Evidence

The quality of the included studies was evaluated using ROBINS-1 [24] and the Cochrane risk of bias assessment tool (version 2) [25] for non-randomized and randomized studies, respectively. The certainty of evidence was also appraised in line with the GRADE (Grading of Recommendations, Assessment, Development, and Evaluations) approach [26]. Two coauthors conducted the judgements independently, resolving any disagreements through mutual agreement.

### 2.6. Statistical Analysis

One study (Tarafdari 2024) included two arms (letrozole 5-day course and letrozole 10-day course), each treated as a separate intervention for the meta-analysis [16]. The 5-day study was labeled Tarafdari 2024 (5-day course), while the 10-day study was labeled Tarafdari 2024 (10-day course) [16]. The interquartile range and median values were transformed to mean and standard deviation values in line with the recommendations by Wan et al. [27]. Data analysis was conducted using STATA Version 19 Software, with statistical significance set at *p* < 0.05 for all endpoints. The random-effects model [28] was used to summarize the data as mean differences (MD) and risk ratios (RR), along with 95% confidence intervals (CI). Between-study heterogeneity was assessed using the I^2^ statistic with a threshold of >50% [29]. Additionally, leave-one-out sensitivity analyses were performed to evaluate the robustness of the results by sequentially excluding one study at a time and recalculating the summary effect sizes for the remaining RCTs. Publication bias evaluation was not completed as the number of included studies per outcome was fewer than the minimum recommended (n = 10) [30].

## 3. Results

### 3.1. Summary of Literature Search and Included Studies

**Figure 1:**
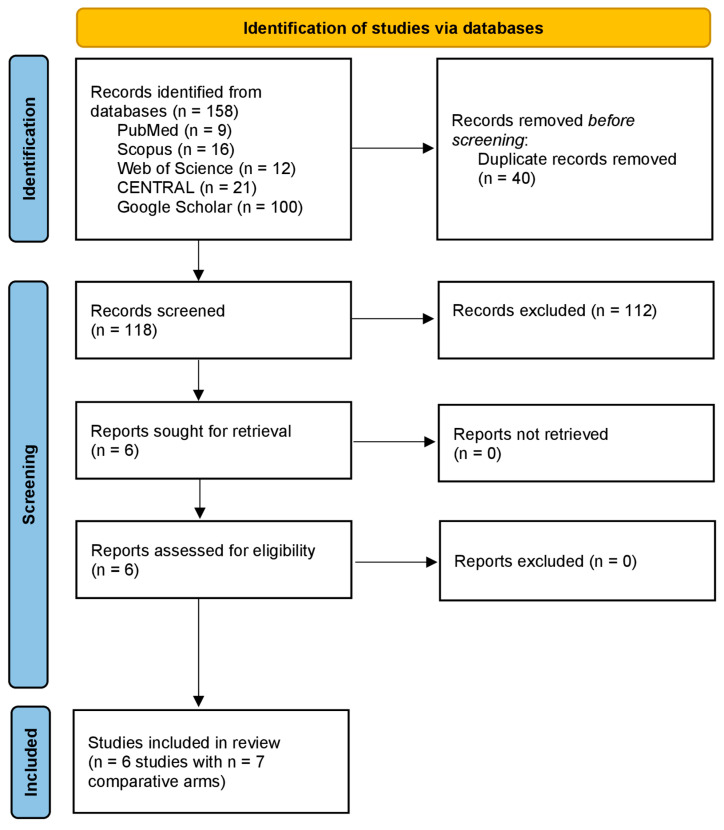
The PRISMA flow diagram for literature search and study selection.

The PRISMA flow diagram illustrating the literature search and study selection process is shown in Figure 1. A total of 158 records were identified through database searches, including PubMed (n = 9), Scopus (n = 16), Web of Science (n = 12), CENTRAL (n = 21), and Google Scholar (n = 100). After removing 40 duplicate records, 118 records remained for screening. Of these, 112 were excluded, and six reports were sought for retrieval and assessed for eligibility. No reports were excluded or unretrieved. Ultimately, six studies comprising seven arms and 260 patients (letrozole = 130, methotrexate = 130) were included in the analysis.

Table 1 provides a summary of the included studies, which consisted of three randomized controlled trials (RCTs) and three non-randomized prospective cohort studies. These studies were conducted between 2020 and 2024 in Egypt (n = 4), Iran (n = 1), and Poland (n = 1). The study arms were relatively small, ranging from 10 to 43 patients. Additionally, most studies used letrozole at a dose of 2.5 mg twice daily for 10 days, while methotrexate was typically given as a single intramuscular injection (1 mg/kg or 50 mg/m^2^ body surface area). Patient ages ranged from the mid-20s to early 30s, with similar BMI values and ectopic pregnancy sizes, all under 4 cm. Baseline HCG levels were generally below 3000 mIU/mL. Most patients had low parity, and ectopic pregnancies were identified as tubal in four studies and adnexal in two. There were no significant differences in baseline characteristics between the letrozole and methotrexate interventional arms.

### 3.2. Summary of Quality of Included Studies

**Figure 2:**
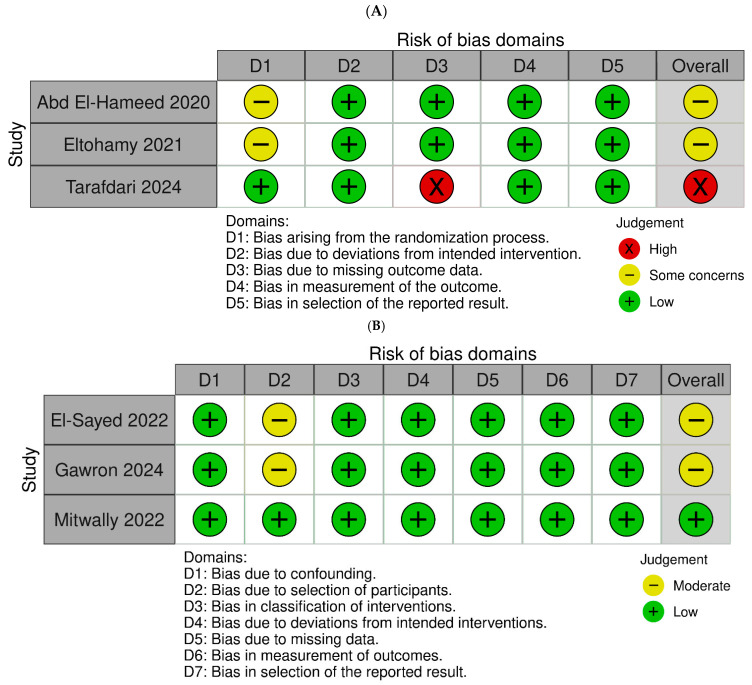
Quality (risk of bias) assessment of the included studies: (**A**) randomized controlled trials and (**B**) non-randomized prospective cohort studies [13,14,15,16,17,18].

Figure 2 presents the risk of bias assessment for the included studies. For the RCTs (Figure 2A), the overall risk of bias was rated as “some concerns” in two studies [13,17] and “high risk” [16] in one study. Two RCTs [13,17] did not clearly report details of the randomization process and allocation concealment, leading to a judgment of “some concerns” for that domain. In one RCT, there was an unbalanced loss of participants during the study, resulting in a “high risk” rating for the domain of missing outcome data [16]. For the non-randomized prospective cohort studies (Figure 2B), the overall risk of bias was assessed as “moderate risk” in two studies [15,18] and “low risk” [14] in one. Two non-RCTs [15,18] did not specify whether consecutive patient recruitment was performed, leading to “some concerns” for the domain related to participant selection.

### 3.3. Meta-Analysis of Primary Outcome

**Figure 3:**
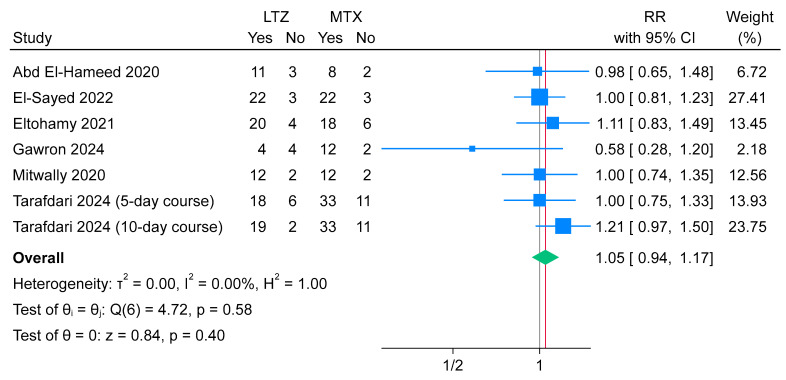
Forest plot meta-analysis of the success rate of ectopic pregnancy treatment [13,14,15,16,17,18].

The success rate of EP treatment did not differ significantly between the letrozole and methotrexate groups (n = 7 arms; RR = 1.05; 95% CI: [0.94, 1.17]; *p* = 0.40). The pooled analysis showed no heterogeneity (I^2^ = 0%, *p* = 0.58) (Figure 3). No publication bias was detected, as indicated by the relative symmetry of the funnel plots and a non-significant Egger’s regression test (*p* = 0.1273; Supplementary Figure S1).

### 3.4. Meta-Analysis of Secondary Outcomes

**Figure 4:**
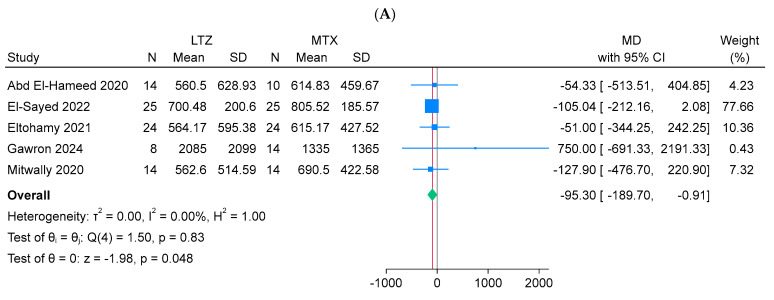

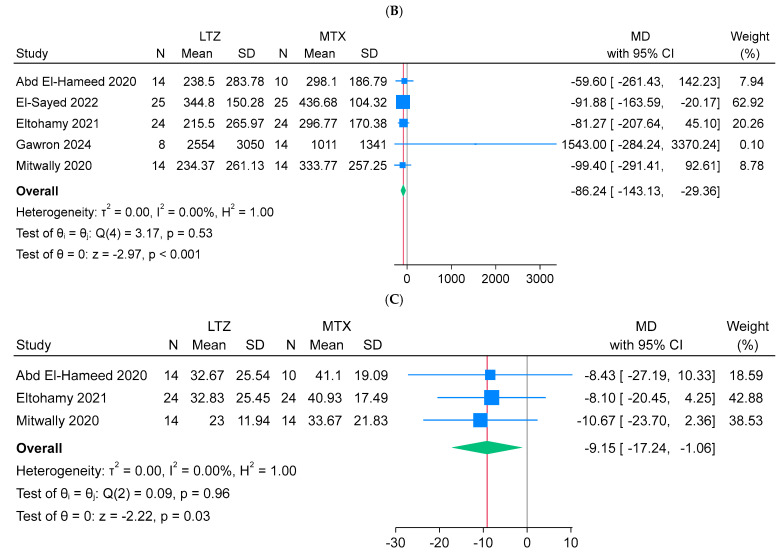
Forest plot meta-analysis of beta-human chorionic gonadotropin levels at different time points following ectopic pregnancy treatment [13,14,15,17,18]: (**A**) Day 4, (**B**) Day 7, and (**C**) Day 14.

From a statistical point of view, the letrozole group showed significantly lower mean β-hCG levels compared to the methotrexate group on day 4 (n = 5 arms; MD = −95 mIU/mL; 95% CI: [−189.7, −0.91]; *p* = 0.048), day 7 (n = 5 arms; MD = −86.24 mIU/mL; 95% CI: [−143.1, −29.36]; *p* < 0.001), and day 14 (n = 3 arms; MD = −9.15 mIU/mL; 95% CI: [−17.24, −1.06]; *p* = 0.03). All pooled results were homogeneous (I^2^ = 0%, *p* > 0.1) (Figure 4A–C).

**Figure 5:**
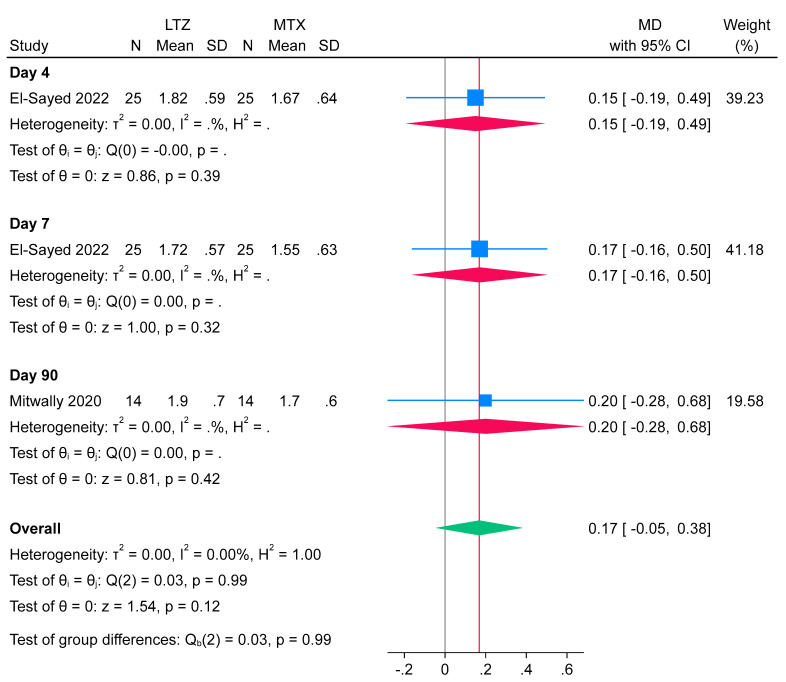
Forest plot meta-analysis of anti-Mullerian hormone (AMH) levels at different time points following ectopic pregnancy treatment [14,18].

AMH levels post-treatment were reported at different time points—day 4, day 7, and day 90—each assessed by a single study. The results, based on the combined data across these time points, showed no significant difference between the letrozole and methotrexate groups (n = 3 arms; MD = 0.17 ng/mL; 95% CI: [−0.05, 0.38]; *p* = 0.99). The pooled analysis demonstrated no heterogeneity (I^2^ = 0%, *p* = 0.99). Results at the individual time points also showed no significant differences between the groups (Figure 5).

**Figure 6:**
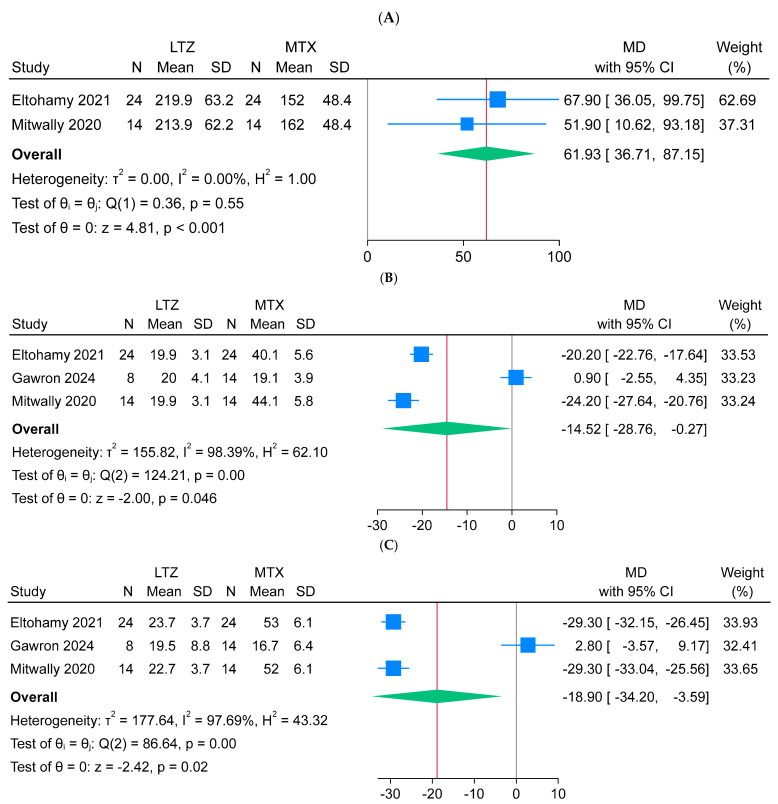
Forest plot meta-analysis of blood parameter levels at 7 days following ectopic pregnancy treatment [13,14,15]: (**A**) platelets, (**B**) aspartate transaminase, and (**C**) alanine transaminase .

Regarding adverse effects, letrozole treatment was favorably associated with a significantly higher platelet count (n = 2 arms; MD = 61.93 × 10^9^/L; 95% CI: [36.71, 87.15]; *p* < 0.001) compared to methotrexate treatment, and the pooled analysis was homogeneous (I^2^ = 0%, *p* = 0.55). In addition, letrozole treatment was also favorably associated with a significant reduction in aspartate transaminase (AST) levels (n = 3 arms; MD = −14.52 U/L; 95% CI: [−28.7, −0.27]; *p* = 0.046) and alanine transaminase (ALT) levels (n = 3 arms; MD = −18.9 U/L; 95% CI: [−34.2, −3.59]; *p* = 0.02). The pooled analyses for AST and ALT were heterogeneous (I^2^ = 98%, *p* < 0.001) (Figure 6A–C). In an attempt to explore the source of heterogeneity, the Galbraith plot did not reveal any clear outliers or identifiable causes for the heterogenous liver transaminase outcomes (Supplementary Figure S2A,B).

### 3.5. Summary of Sensitivity Analysis and Certainty of Evidence

Supplementary Figure S3A–G present the results of the sensitivity analyses. The primary outcome (the success rate of EP treatment) demonstrated stability, as the omission of individual studies did not significantly affect the pooled RR. Conversely, all secondary outcomes showed instability, with the exclusion of individual studies leading to significant changes in the MD estimates. Table 2 presents the summary of certainty of evidence according to the GRADE approach, with all outcomes rated as ‘very low’.

## 4. Discussion

We conducted a meta-analysis of six studies (three RCTs and three non-randomized cohorts) comprising seven study arms, comparing letrozole monotherapy (n = 130) to methotrexate (n = 130) for the treatment of ectopic pregnancy. Risk of bias was rated as low in one study, moderate in four, and high in one. No significant difference was observed in treatment success rates, and sensitivity analysis supported the robustness of this finding. Although publication bias was not detected, the small number of included studies limits the reliability of this assessment. Clinically, the findings suggest that letrozole may offer similar effectiveness to methotrexate; however, due to the very low certainty of evidence and limited sample sizes, these results should be interpreted with caution and do not establish equivalence. Letrozole was associated with significantly lower β-hCG levels at multiple time points, though these differences were not clinically meaningful. Typically, a relative decline of ≥10–15% is considered more informative than an absolute mIU/mL difference [31]. AMH levels were similar between groups. Letrozole also demonstrated a more favorable safety profile, with higher platelet counts and lower liver enzyme levels. Overall, the certainty of evidence was rated as “very low” according to GRADE, reflecting we have very little confidence in the effect estimate; the true effect is likely to be substantially different from the reported estimate.

The results of the primary outcome should be interpreted with caution due to notable imprecision. Most included studies had relatively small sample sizes per treatment arm, and the pooled effect estimates were accompanied by wide confidence intervals. This imprecision reduces the certainty of the evidence and limits the strength of the conclusions that can be drawn. These findings underscore the need for larger, high-quality RCTs to better assess the comparative efficacy and safety of letrozole versus methotrexate in the treatment of EP. Moreover, the high heterogeneity observed for liver enzyme outcomes (AST and ALT), as indicated by I^2^ values of 98%, warrants careful consideration. This substantial variability may be attributed to differences in study design, as two of the included studies were non-randomized while one was an RCT, potentially introducing methodological inconsistencies. Additionally, variation in dosing regimens, follow-up durations, and baseline patient characteristics may have contributed to the observed heterogeneity. While subgroup analysis could be a useful approach to explore these differences, it was not feasible in this review due to the limited number of available studies reporting on these outcomes. As such, the pooled estimates for AST and ALT should be interpreted with caution, and future studies employing standardized protocols are needed to better clarify the impact of letrozole and methotrexate on liver function in the management of EP.

Across the included studies, AMH was measured at varying time points following treatment, specifically on day 4, day 7, and day 90, with each time point represented by only one study. This lack of uniformity and the limited number of data points led us to consider AMH as a secondary outcome. Consequently, the inconsistent timing and sparse reporting hinder meaningful comparison and interpretation of ovarian reserve effects following treatment. We acknowledge it as a limitation in our study and underscore the need for future research to standardize AMH measurement time points and further investigate its role as a biomarker for ovarian function after medical management of EP.

Surgical treatment for EP is definitive but invasive, with associated risks and complications such as infection, hemorrhage, damage to the fallopian tubes, and adhesions [2,3,4]. Medical therapy is often recommended for stable, unruptured EP cases, with methotrexate being the first-line agent [5]. However, methotrexate has several side effects and contraindications that can deter its utilization, including active liver disease, renal impairment, peptic ulcer disease, folate deficiency, and immunosuppression [7]. Additionally, methotrexate is generally not recommended for use in EP when β-HCG levels exceed 5000 IU/L because higher β-HCG levels are associated with lower success rates for medical treatment [32,33].

Letrozole is commonly used to treat various gynecological disorders, thanks to its effective anti-estrogenic properties, high tolerability, low cost, and minimal side effects. It has been shown to reduce the size of uterine myomas [34,35], induce ovulation in cases of polycystic ovarian syndrome [36], and help reduce the pain and recurrence of endometriosis [37]. This meta-analysis further broadens the potential applications of letrozole, highlighting its use in the medical management of EP.

Recent studies have investigated the use of letrozole as an alternative pharmacotherapy for EP [13,14,15,16,17,18]. The rationale for using letrozole in EP treatment lies in its ability to inhibit the aromatase enzyme, thereby reducing levels of placental estrogen, progesterone, and VEGF [9,10,11,12,38]. Mechanistically, letrozole reduces the expression of steroid receptors, including those for estrogen and progesterone, leading to decreased receptor activity. Additionally, the reduction in estrogen levels likely disrupts the physiological functions of progesterone necessary to sustain pregnancy. Letrozole also lowers VEGF, an angiogenic factor crucial for implantation and placentation in EP. These mechanisms collectively support the clinical use of letrozole in the medical management of EP.

Compared to methotrexate, letrozole appears to have a safer profile, making it a potentially attractive option for the medical management of EP. One included study by Alabiad et al. reported that a 10 mg letrozole regimen resulted in a higher rate of EP resolution compared to a 5 mg regimen (85% vs. 65%), with no serious side effects observed in either group [38]. However, this finding is based on a single study and, in our meta-analysis, subgroup analysis by dose was not possible since nearly all study arms used the 5 mg dose. Additionally, the 10 mg group experienced a transient increase in liver enzymes, which resolved spontaneously without clinical consequences. While these preliminary results suggest a possible dose-dependent effect, further well-designed studies are needed to confirm the optimal dosing strategy and safety profile of letrozole in EP.

Our study has several key strengths that deserve highlighting. It is the most comprehensive meta-analysis to date comparing the efficacy of letrozole versus methotrexate in the medical management of EP. We conducted an extensive search across five databases to ensure the inclusion of all relevant studies. Additionally, we reported multiple endpoints to provide a thorough understanding of the findings. The robustness of our conclusions was further supported by rigorous analyses, including sensitivity analyses and grading of the certainty of evidence—methods that have not been employed in previous studies [19,20].

Despite its strengths, our study has several limitations that should be acknowledged. The relatively small number of studies included, along with their limited sample sizes, represents a significant constraint. Additionally, some endpoints showed heterogeneity, which may be attributed to variations in perioperative factors such as patient characteristics, clinical features of EP, and routes of drug administration. These factors could influence the conclusions drawn from the pooled data. Given that the number of studies was limited (n < 10), we did not assess publication bias [30]. Furthermore, the majority of included studies were conducted in Egypt, which may limit the generalizability of our findings to other populations due to potential regional differences in clinical practices, patient characteristics, and healthcare settings. Lastly, some studies had risk of bias in domains of randomization, allocation concealment, and missing outcome due to loss of follow-up, potentially affecting the reliability of the conclusions.

Given the limitations of our study, future research should focus on conducting large, multicenter, and well-controlled RCTs comparing the efficacy and safety of letrozole and methotrexate in the medical management of EP. To ensure sufficient statistical power and address the imprecision noted in current evidence, sample size calculations should be based on clinically meaningful differences in treatment success rates, ideally enrolling several hundred patients per arm. Prospective studies should also examine the effectiveness of these drugs across different patient subgroups, such as varying age groups, baseline β-hCG levels, and EP locations, to identify populations who may benefit most. Additionally, further research is warranted to explore the dose-dependent effects of letrozole and establish the optimal dosing regimen. Standardized measurement of ovarian reserve markers, particularly AMH at specific, consistent time points post-treatment, should be incorporated to better assess the impact of medical therapies on fertility. It would also be valuable to compare letrozole with other active agents, including misoprostol, mifepristone, or potassium chloride. Finally, studies investigating the potential benefit of combining letrozole with methotrexate versus methotrexate alone could provide insight into improved treatment outcomes and reduced surgical intervention rates.

## 5. Conclusions

Letrozole may be a promising alternative to methotrexate for the medical management of EP. This meta-analysis indicates that letrozole provides comparable efficacy to methotrexate while offering a better safety profile, highlighting its potential as a safer treatment option. However, the certainty of the current evidence is very low due to risk of bias and imprecision, and these findings should be interpreted with caution. Therefore, further high-quality studies are urgently needed to establish the clinical utility of letrozole in EP management.

## Figures and Tables

**Table 1:** The baseline characteristics of the included studies.

Study Identifier	Country	Study Design	Study Arms	n	Intervention	Age (Year)	BMI (kg/m^2^)	EP Size (cm)	Pre-HCG Titer (mIU/mL)	Parity	EP Site
Abd El-Hammed 2020 [17]	Egypt	RCT	LTZ	14	2.5 mg tablet, BID, for 10 days	26 ± 4	21 ± 2	3.3 ± 0.6	865 (486–1416)	NR	Adnexal
			MTX	10	1 mg/kg, IM, one injection	28 ± 7	22 ± 3	3.1 ± 0.8	1328 (735–1647)	NR	
El-Sayed 2022 [18]	Egypt	Non-RCT	LTZ	25	2.5 mg tablet, BID, for 10 days	28 ± 3	28 ± 2	NR	1134 ± 368	2 ± 0.82	Fallopian tube
			MTX	25	1 mg/kg, IM, one injection	30 ± 4	29 ± 2	NR	1119 ± 348	1.6 ± 0.7	
Eltohamy 2021 [13]	Egypt	RCT	LTZ	24	2.5 mg tablet, BID, for 10 days	27 ± 12	22 ± 2	3.2 ± 0.6	856 ± 123	NR	Fallopian tube
			MTX	24	50 mg/m^2^ BSA, IM, one injection			3.0 ± 0.8	1331 ± 235	NR	
Gawron 2024 [15]	Poland	Non-RCT	LTZ	8	2.5 mg tablet, BID, for 10 days	32 ± 5	NR	1.63 ± 0.53	1464 ± 1219	NR	Fallopian tube
			MTX	14	100 mg, IV, one injection	31 ± 4	NR	1.32 ± 0.35	1189 ± 828	NR	
Mitwally 2020 [14]	Egypt	Non-RCT	LTZ	14	2.5 mg tablet, BID, for 10 days	26 ± 4	26 ± 4	NR	1065 (492–1438)	NR	Fallopian tube
			MTX	14	50 mg/m^2^ BSA, IM, one injection	26 ± 5	27 ± 5	NR	1415 (710–1722)	NR	
Tarafdari 2024 [16]	Iran	RCT	LTZ	24	2.5 mg tablet, TID, for 5 days	32 ± 6	NR	2.54 ± 0.68	944.4 ± 802.9	0.5 ± 0.7	Adnexal
			LTZ	21	2.5 mg tablet, BID, for 10 days	34 ± 4	NR	2.59 ± 0.8	780.7 ± 595.2	0.8 ± 0.9	
			MTX	43	50 mg/m^2^ BSA, IM, one injection	31 ± 6	NR	2.8 ±1.47	990.6 ± 754.0	1.2 ± 1.1	

BID: twice daily; BMI: body mass index; BSA: body surface area; EP: ectopic pregnancy; IM: intramuscular injection; IV: intravenous injection; LTZ: letrozole; MTX: methotrexate; n: number; non-RCT: non-randomized controlled trial; RCT: randomized controlled trial; TID: thrice daily. Age and body mass index were reported as mean ± standard deviation and rounded to the nearest whole number. EP size was reported as mean ± standard deviation. Pre-HCG titer was as mean ± standard deviation or median (interquartile range). Parity was reported as mean ± standard deviation.

**Table 2:** Summary of the quality of evidence according to the GRADE approach.

Endpoint	Certainty Assessment							Overall Certainty
	Number of Arms	Study Design	Risk of Bias *	Inconsistency **	Indirectness	Imprecision ***	Publication Bias	
Success rate of EP treatment	7	RCT and non-RCT	Very serious	Not serious	Not serious	Serious	Strongly suspected	⨁◯◯◯ Very low
Beta-hCG level on day 4	5	RCT and non-RCT	Very serious	Not serious	Not serious	Serious	Strongly suspected	⨁◯◯◯ Very low
Beta-hCG level on day 7	5	RCT and non-RCT	Very serious	Not serious	Not serious	Serious	Strongly suspected	⨁◯◯◯ Very low
Beta-hCG level on day 14	3	RCT and non-RCT	Very serious	Not serious	Not serious	Serious	Strongly suspected	⨁◯◯◯ Very low
AMH level	3	RCT and non-RCT	Very serious	Not serious	Not serious	Serious	Strongly suspected	⨁◯◯◯ Very low
Platelet count on day 7	2	RCT and non-RCT	Very serious	Not serious	Not serious	Serious	Strongly suspected	⨁◯◯◯ Very low
AST level on day 7	3	Non-RCT	Very serious	Serious	Not serious	Serious	Strongly suspected	⨁◯◯◯ Very low
ALT level on day 7	3	Non-RCT	Very serious	Serious	Not serious	Serious	Strongly suspected	⨁◯◯◯ Very low

ALT: alanine transaminase; AMH: anti-Mullerian hormone; AST: aspartate transaminase; EP: ectopic pregnancy; hCG: human chorionic gonadotrophin. * Risk of bias was judged as very serious because most studies had some concerns regarding the randomization process and allocation concealment. ** Inconsistency was judged as serious when the between-study heterogeneity was significant (I^2^ statistic > 50%). *** Imprecision was judged as serious because of the small sample size.

## Data Availability

All data are available within the manuscript and its supplemental files.

## Supplementary Materials

The following supporting information can be downloaded at: https://www.mdpi.com/article/10.3390/jcm14186523/s1, Figure S1: Publication bias analysis of the success rate of ectopic pregnancy treatment; Figure S2: Galbraith plots for heterogeneous outcomes: [A] aspartate transaminase levels and [B] alanine transaminase levels, seven days post-treatment; Figure S3: Leave-one-out sensitivity analysis was performed for the following outcomes: [A] success rate of ectopic pregnancy treatment, [B] beta-human chorionic gonadotropin levels 4 days post-treatment, [C] beta-human chorionic gonadotropin levels 7 days post-treatment, [D] beta-human chorionic gonadotropin levels 14 days post-treatment, [E] platelet counts 7 days post-treatment, [F] aspartate transaminase levels 7 days post-treatment, and [G] alanine transaminase levels 7 days post-treatment; Table S1: The search strategy used in all databases.
