# Supplementary material for: Efficacy of Monotherapy Letrozole Versus Methotrexate for the Management of Ectopic Pregnancy: A Systematic Review and Meta-Analysis of Comparative Studies
Source: J Clin Med. 2025 Sep 17;14(18):6523. doi: 10.3390/jcm14186523 (PMC12470578; doi:10.3390/jcm14186523)
Supplement: Supplementary file 1 [file jcm-14-06523-s001.zip › Supplementary Figures_09-06-25.pdf]

Supplementary Figure S1. Publication bias analysis of the success rate of ectopic pregnancy treatment.

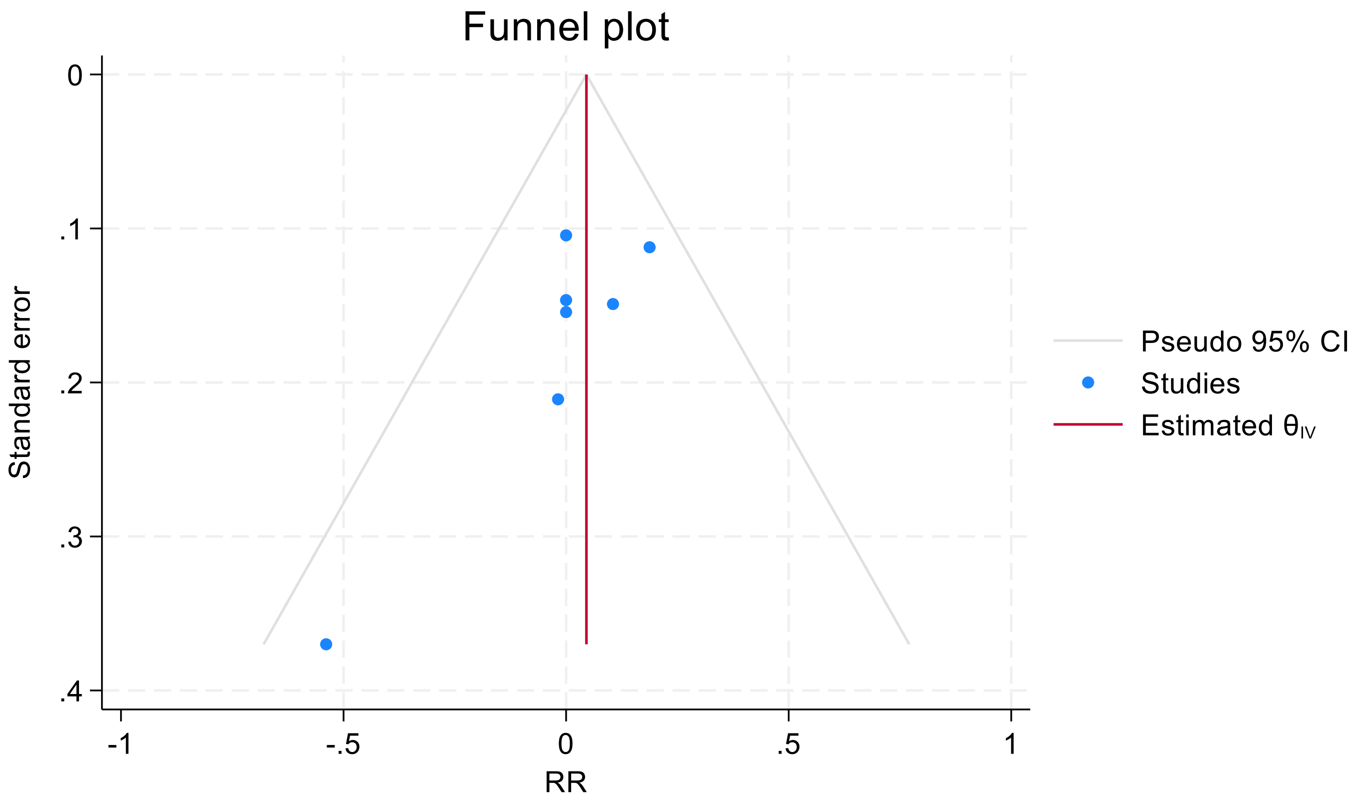

Supplementary Figure S2. Galbraith plots for heterogeneous outcomes: **[A]** aspartate transaminase levels and **[B]** alanine transaminase levels, seven days post-treatment.

[A]

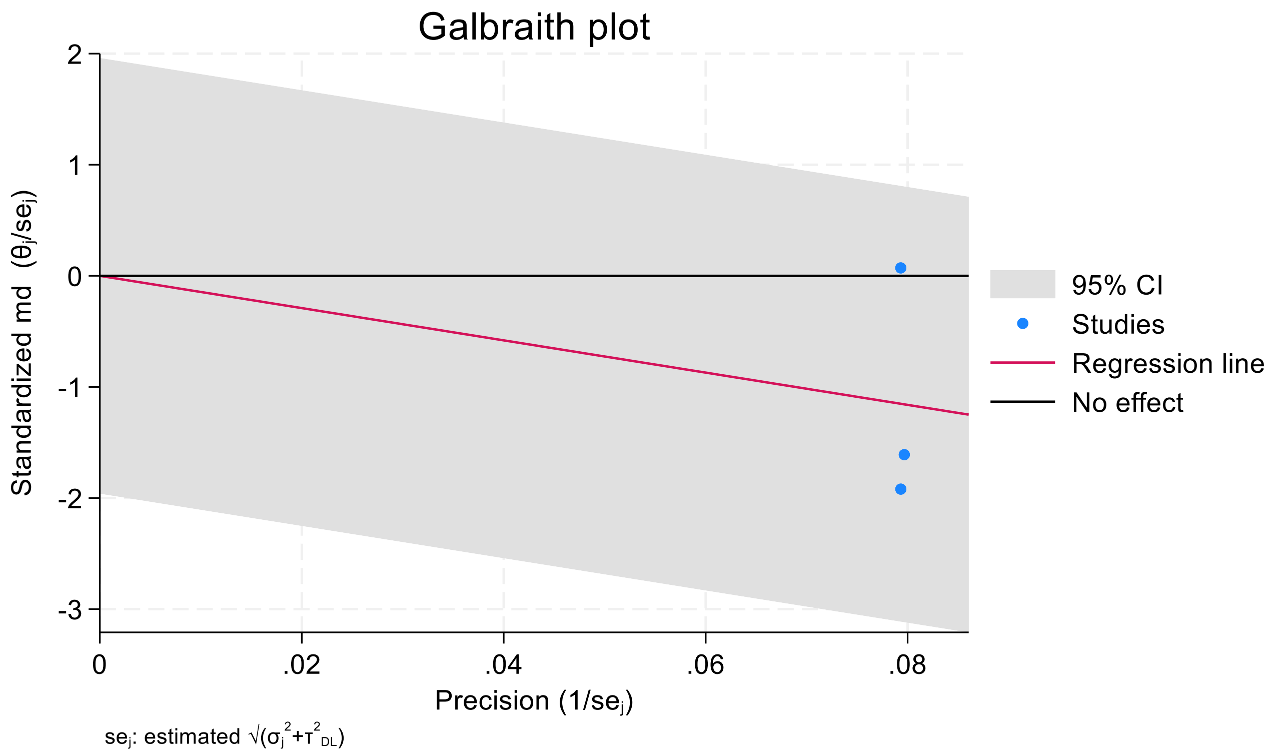

[B]

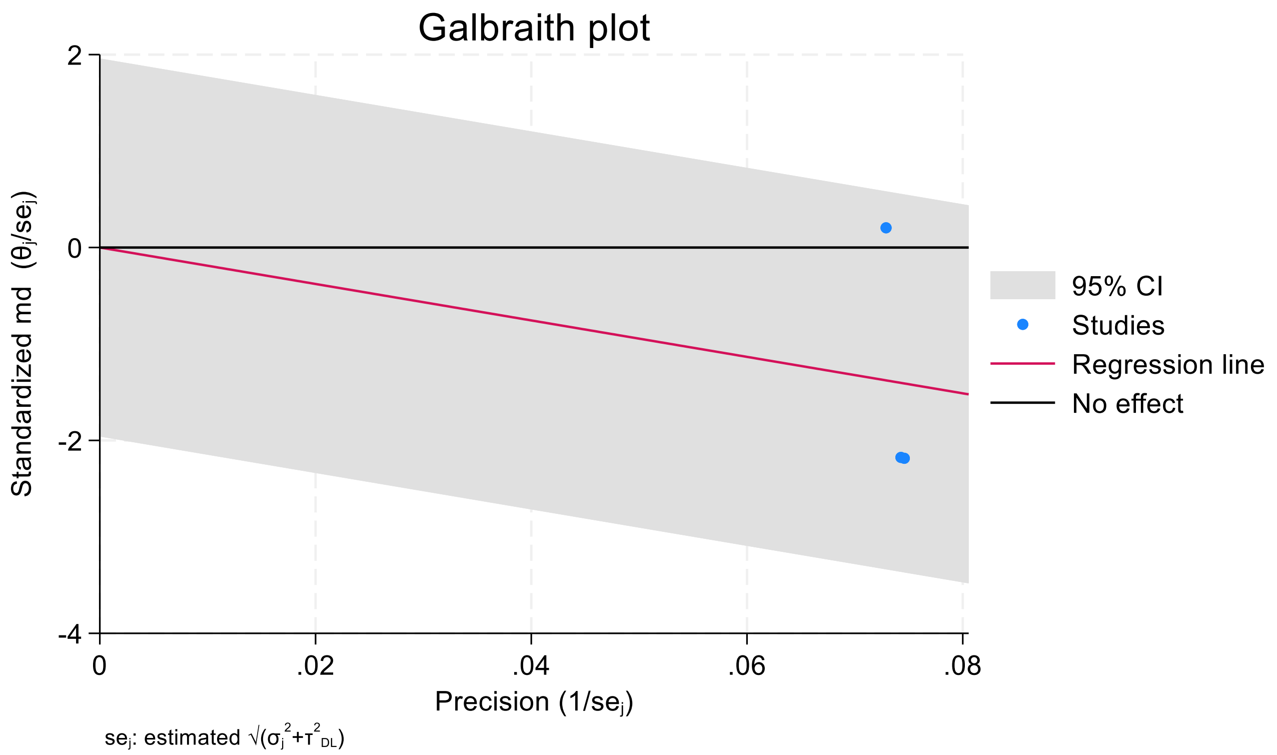

Supplementary Figure S3. Leave-one-out sensitivity analysis was performed for the following outcomes: **[A]** success rate of ectopic pregnancy treatment, **[B]** beta-human chorionic gonadotropin levels 4 days post-treatment, **[C]** beta-human chorionic gonadotropin levels 7 days post-treatment, **[D]** beta-human chorionic gonadotropin levels 14 days post-treatment, **[E]** platelet counts 7 days post-treatment, **[F]** aspartate transaminase levels 7 days post-treatment, and **[G]** alanine transaminase levels 7 days post-treatment.

[A]

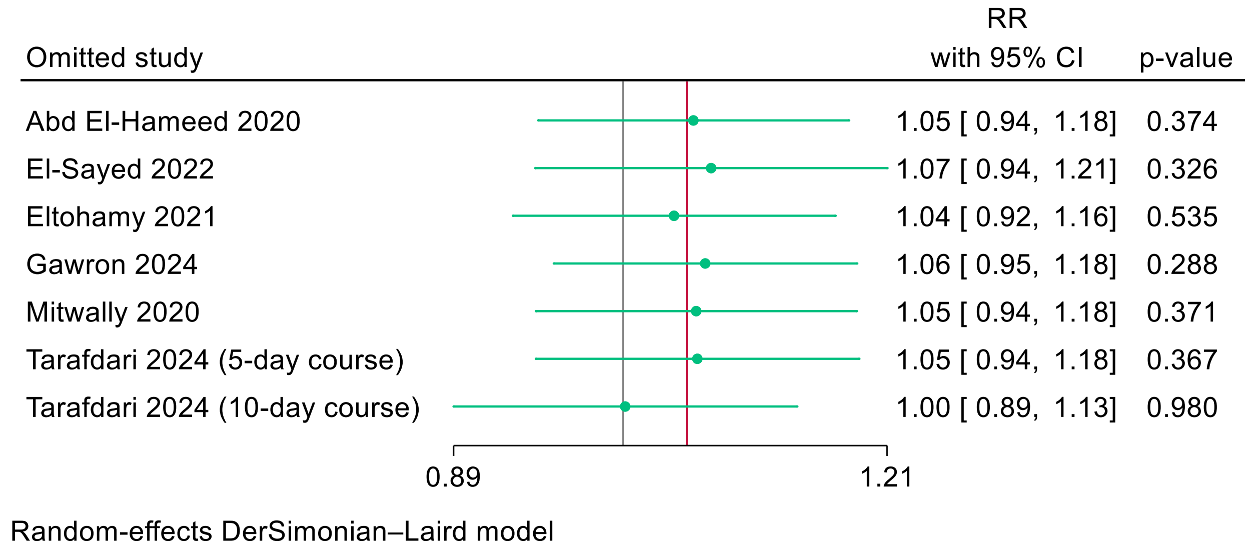

[B]

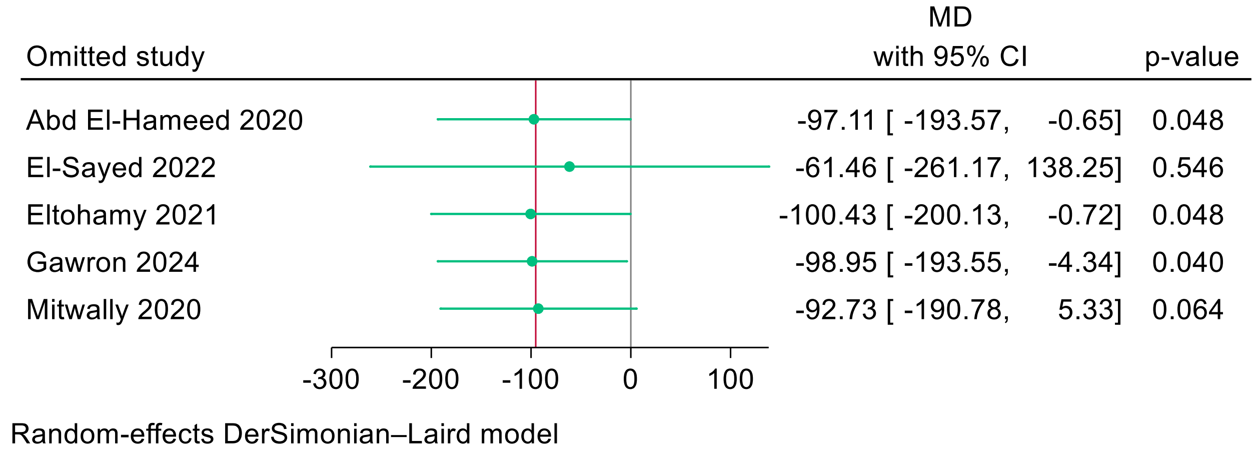

[C]

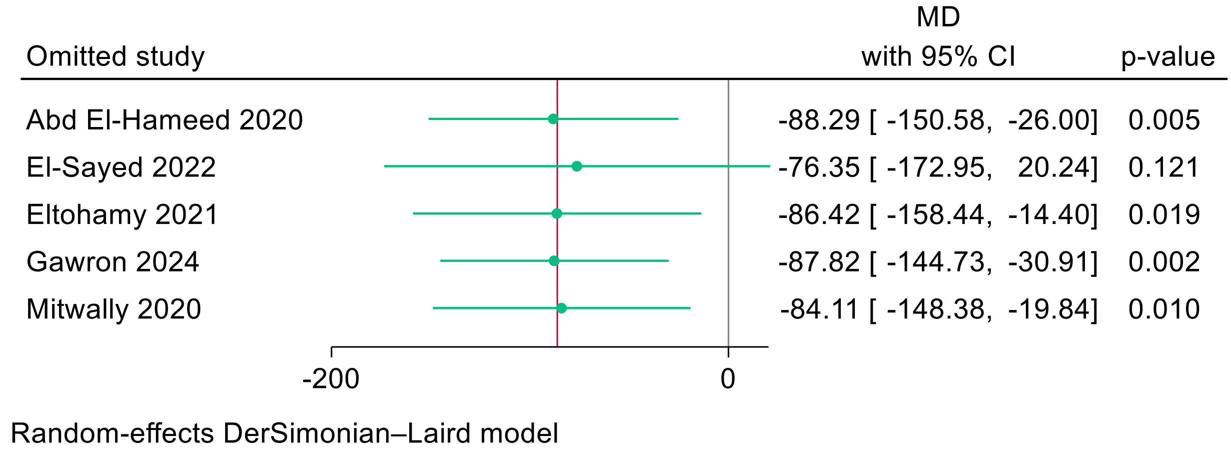

[D]

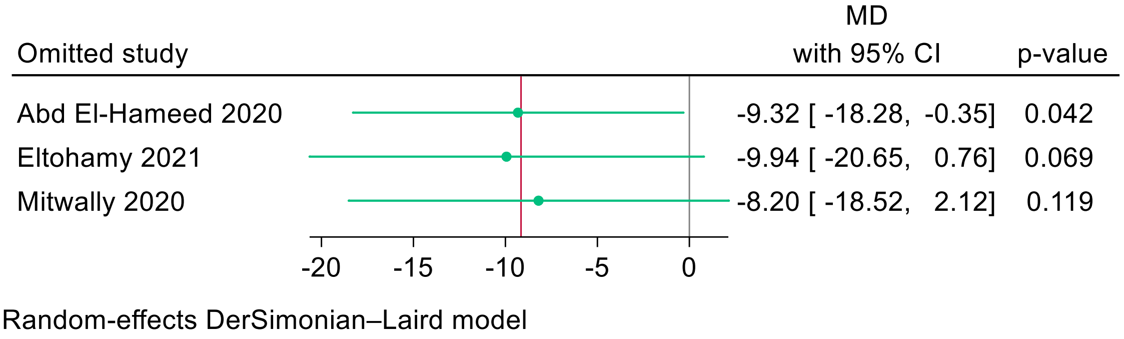
Omitted study
MD
with 95% CI
p-value
Abd El-Hameed 2020
-9.32 [-18.28, -0.35]
0.042
Eltohamy 2021
-9.94 [-20.65, 0.76]
0.069
Mitwally 2020
-8.20 [-18.52, 2.12]
0.119
-20
-15
-10
-5
0
Random-effects DerSimonian–Laird model

[E]

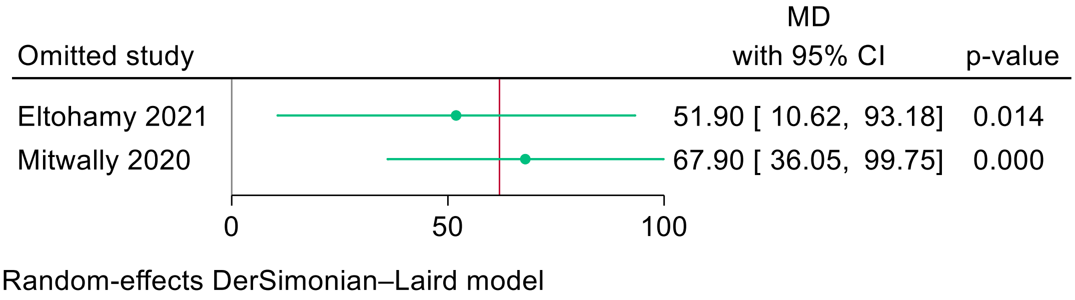
Omitted study
MD
with 95% CI
p-value
Eltohamy 2021
51.90 [10.62, 93.18]
0.014
Mitwally 2020
67.90 [36.05, 99.75]
0.000
0
50
100
Random-effects DerSimonian–Laird model

[F]

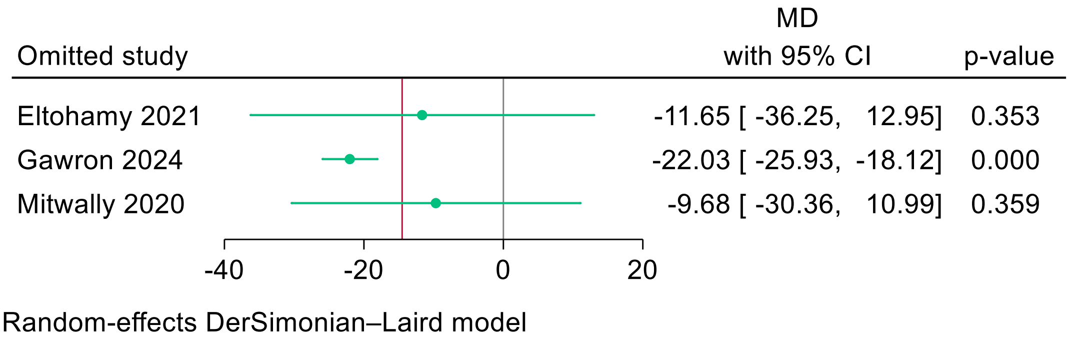
Omitted study
MD
with 95% CI
p-value
Eltohamy 2021
-11.65 [-36.25, 12.95]
0.353
Gawron 2024
-22.03 [-25.93, -18.12]
0.000
Mitwally 2020
-9.68 [-30.36, 10.99]
0.359
-40
-20
0
20
Random-effects DerSimonian–Laird model

[G]

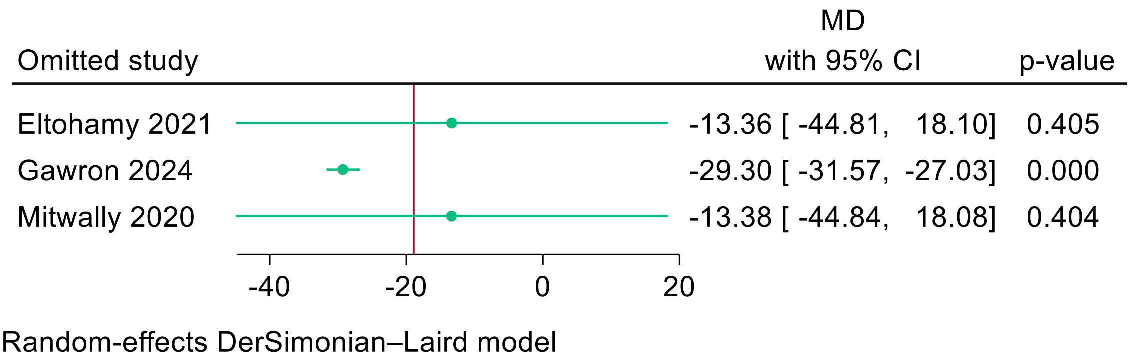
Omitted study
MD
with 95% CI
p-value
Eltohamy 2021
-13.36 [-44.81, 18.10]
0.405
Gawron 2024
-29.30 [-31.57, -27.03]
0.000
Mitwally 2020
-13.38 [-44.84, 18.08]
0.404
-40
-20
0
20
Random-effects DerSimonian–Laird model
