# Supplementary material for: Efficacy of Monotherapy Letrozole Versus Methotrexate for the Management of Ectopic Pregnancy: A Systematic Review and Meta-Analysis of Comparative Studies
Source: J Clin Med. 2025 Sep 17;14(18):6523. doi: 10.3390/jcm14186523 (PMC12470578; doi:10.3390/jcm14186523)
Supplement: Supplementary file 1 [file jcm-14-06523-s001.zip › Supplementary Table 1_09-06-25.pdf]

Supplementary Table S1. The search strategy used in all databases.

**PubMed**<br>All Fields: (letrozole OR femara) AND (methotrexate OR amethopterin) AND (ectopic OR extrauterine OR tubal) AND (pregnancy)
**Scopus**<br>TITLE-ABS-KEY ((letrozole OR femara) AND (methotrexate OR amethopterin) AND (ectopic OR extrauterine OR tubal) AND (pregnancy))
**Web of Science**<br>All Fields: (letrozole OR femara) AND (methotrexate OR amethopterin) AND (ectopic OR extrauterine OR tubal) AND (pregnancy)
**Embase**<br>Broad search: ('letrozole'/exp OR letrozole OR 'femara'/exp OR femara) AND ('methotrexate'/exp OR methotrexate OR 'amethopterin'/exp OR amethopterin) AND (ectopic OR extrauterine OR tubal) AND ('pregnancy'/exp OR pregnancy)
**Cochrane Central Register of Controlled Trials (CENTRAL)**<br>Title Abstract Keyword: (letrozole OR femara) AND (methotrexate OR amethopterin) AND (ectopic OR extrauterine OR tubal) AND (pregnancy)
**Google Scholar**<br>All Fields: (letrozole OR femara) AND (methotrexate OR amethopterin) AND (ectopic OR extrauterine OR tubal) AND (pregnancy)
